# An Examination of Patients and Caregivers on Reddit Navigating Brain Cancer: Content Analysis of the Brain Tumor Subreddit

**DOI:** 10.2196/35324

**Published:** 2022-06-22

**Authors:** Sanidhya D Tripathi, Pearman D Parker, Arpan V Prabhu, Kevin Thomas, Analiz Rodriguez

**Affiliations:** 1 Department of Biomedical Engineering University of Arkansas Fayetteville, AR United States; 2 College of Nursing University of Arkansas for Medical Sciences Little Rock, AR United States; 3 Department of Radiation Oncology University of Arkansas for Medical Sciences Little Rock, AR United States; 4 Department of Neurosurgery University of Arkansas for Medical Sciences Little Rock, AR United States

**Keywords:** brain tumor, internet, social media, Reddit, cancer, emotional support, self-management

## Abstract

**Background:**

Occurring in up to 40% of all patients with cancer, the incidence of brain tumors has caused limited survival, a high psychosocial burden, and an increase in the loss of decision-making capability for the unique population. Although specific symptoms depend on the type of brain tumor, a clinical team of physicians, nurses, and other individuals commonly assist patients and their caregivers with how to tackle the upcoming challenges of their diagnosis. Despite the support from clinical team members, many patients and caregivers may still seek outside support through social media to process their emotions and seek comfort outside of the clinical setting. Specifically, online resources such as Reddit are used where users are provided with the anonymity they need to show their true behavior without fear of judgment. In this study, we aimed to examine trends from Reddit discussion threads on brain tumors to identify areas of need in patient care.

**Objective:**

Our primary aims were to determine the type of Reddit user posting, classify the specific brain tumors that were the subject of the posts, and examine the content of the original posts.

**Methods:**

We used a qualitative descriptive design to understand patients’ and caregivers’ unmet and met needs. We selected posts from the top-rated 100 posts from the r/braincancer subreddit from February 2017 to June 2020 to identify common themes using content analysis.

**Results:**

The qualitative content analysis revealed how Reddit users primarily used the forum as a method to understand and process the emotions surrounding a brain tumor diagnosis. Three major topic areas from content analysis emerged as prominent themes, including (1) harnessing hope, (2) moving through the grief process, and (3) expressing gratitude toward other Reddit users. Most of the authors of the posts were patients with brain tumors (32/88, 36%) who used Reddit as a reflective journaling tool to process the associated emotions of a challenging diagnosis.

**Conclusions:**

This study shows the potential of Reddit to serve as a unique group therapy platform for patients affected by brain tumors. Our results highlight the support provided by the Reddit community members as a unique mechanism to assist cancer survivors and caregivers with the emotional processing of living with brain tumors. Additionally, the results highlight the importance of recommending Reddit as a therapeutic virtual community and the need for implementing online resources as a part of a health care professional’s repertoire to understand the level of support they can give their patients.

## Introduction

Brain metastases remain the most common intracranial tumors in adults and can occur in up to 40% of all patients with cancer [1]. With more than 100 histopathologic types of primary central nervous system tumors [2], glioblastoma remains the most common malignant brain tumor in adults and continues to have a grim prognosis [3]. In comparison to other cancers, the potential limited overall survival, the higher psychosocial burden, and the increased likelihood of eventual loss of decision-making capability make patients with brain tumors a unique population. The emotional and physical sequalae following a brain tumor diagnosis can be devastating for patients, caregivers, family members, and friends. This may include dealing with difficult emotions such as sadness or anger and coping with the costs of cancer care with treatments and visits with the medical team. Patient advocacy groups such as the National Coalition for Cancer Survivorship have advocated for the incorporation of more patient-centered communication throughout the cancer care continuum [4].

The clinical team of neurosurgeons, medical and surgical oncologists, radiation oncologists, oncology nurses, and other patient care members guides patients and caregivers throughout the process. Team members assist patients and caregivers with what to expect with treatment and how to prepare for upcoming mental, emotional, and physical challenges. Although specific symptoms depend on the type of brain tumor, common challenges include weakness and balance difficulties along with changes in memory and even personality. Executive functioning can often be significantly impaired, and perception of this decline is often concordant between the patient and caregiver [5,6]. Even though the team members guide patients and caregivers through the process of diagnosis through end-of-life care, patients and caregivers may look for other avenues to express and ultimately process their emotions to make sense of the brain tumor diagnosis. However, these other avenues such as online resources for self-management of living with primary brain cancer have significant gaps in addressing patient and caregiver needs in the areas of rehabilitation, behavioral changes, recurrence, and the transition to palliative care [7].

Patients and caregivers may seek out and use social media to make sense of their illness. These social media platforms can also capture key attitudes and behaviors that are not always reflected in traditional medical surveys [8-11]. The social media sites including Reddit, Instagram, Twitter, and Facebook allow users to share stories, solicit advice, make recommendations, and establish a sense of virtual community. One of these social media sites (Reddit) provides anonymity that can give users a sense of comfort where they are allowed to act in accordance with their natural disposition without fear of judgement [12]. The anonymity combined with virtual storytelling can foster a real therapeutic relationship for patients and caregivers, especially for those affected by life-changing disease processes such as brain tumors [13,14]. In addition to patients and caregivers, key stakeholders such as physicians, patient advocates, patient organizations, and medical researchers use social media sites for the dissemination of information [15-17].

Though these therapeutic virtual relationships can be beneficial for patients and caregivers, health care professionals (HPs) may also be interested in examining the content of these posts to determine if any additional clinical support may be needed. Identifying the category of Reddit user (eg, patient, caregiver, or friend), type of brain tumor, and type of content within the posts may provide HPs with additional knowledge on how their patients are using social media sites like Reddit to potentially cope with their diagnoses. This may help HPs better understand the potential positive role that social media plays in their patients’ lives during their cancer treatment experiences and into palliative care.

Although previous research has drawn information from Reddit to examine various other diseases [18-20], no studies have examined Reddit discussion threads on brain tumors. A thorough content analysis of users’ posts may contribute to a greater understanding of how patients and caregivers use Reddit as a social media platform to discuss and cope with brain tumors. Additionally, determining the category of Reddit users and types of brain tumors identified in the posts may add a greater understanding to specific populations’ needs. Our primary goals were to determine the type of Reddit user posting, classify the specific brain tumors that were the subject of the posts, and examine the content of the original posts.

## Methods

### Overview

The data were obtained from the top-rated 100 posts of the subreddit “r/braincancer” from February 2017 to June 2020. We were able to identify the top-rated posts using the “Top” and “All-time” selection features that sort the subreddit posts from the most upvoted post to the least upvoted post. A post can be upvoted or downvoted by subreddit users as being relevant or recommended to other users, if other users resonate with the content made by the original user who made the post, or to increase visibility of the post to the broader subreddit community. An upvote means the post contributes to the conversation in the subreddit, and a downvote means the post does not contribute [21]. Each user is only granted one vote per post. Subreddit users can then self-select to see only the top-rated posts. All posts that were obtained after applying the filters discussed brain tumors in a variety of contexts.

We extracted data in the posts including title, text, images, usernames, and content. All data was deidentified by removing usernames and then imported into Word (Microsoft Corporation), followed by MAXQDA (VERBI GmbH) for analysis [22]. This software was chosen based on the endorsement of the home institution where the data analysis was conducted. Any posts that were not available at the time of data extraction were not included in the analysis.

Four authors (SDT, PDP, AVP, and AR) agreed upon and set codes to categorize users and types of brain tumors a priori to determine who was posting and which type of brain tumor was being discussed. Two authors (SDT and PDP) then used open coding to reveal the themes of the post content. After identifying the initial list of themes, the two team members met to agree upon the categories to be used for coding. All team members met to discuss the naming of the themes, which were interpretations directly supported by the data. As a final step in the data analysis, we then sorted the themes into categories following Yalom’s [23] group psychotherapeutic factors. This sorting of data was a last step to present the findings in a cohesive manner that resembled the key therapeutic factors in a group setting, and we then applied these factors to a virtual group community. Any discordant coding was resolved and reviewed by an expert in qualitative research.

### Ethical Considerations

This study did not meet the criteria for human participant research review through the University of Arkansas for Medical Sciences Institutional Review Board for protocol #27446. The data were obtained from a publicly available anonymous online forum. Each post included an author’s self-chosen username, and even though these usernames were anonymous, we maintained confidentiality by removing usernames prior to importing the data into the software used for data analysis.

## Results

### Overview

The subreddit “r/braincancer” included a high number of members (n=1900), and the posts covered topics about numerous aspects of brain cancer from treatment to emotional support. The content of the original posts ranged from journal-style storytelling to the sharing of images and uplifting graphics.

Of the top 100 posts, all posts were written by individual users as unique cases. Most of the 88 Reddit users (n=58, 66%) who posted in the “r/braincancer” subreddit were writing about their experience with a patient with a brain tumor. Many of the posts came from users writing about their parent’s (n=32, 36%), spouse’s (n=9, 10%), or children’s (n=9, 10%) experiences with brain tumors. A smaller percentage of Reddit users wrote about their own experiences with brain tumors (n=30, 34%). Only 12 users did not disclose their relationship about who they were writing about in the subreddit thread (see Table 1).

Almost half (n=47, 47%) of the 100 Reddit users directly mentioned glioblastoma in their posts. A smaller amount of Reddit users (n=16, 16%) identified another primary brain tumor that included anaplastic astrocytoma, ganglioglioma, and others. However, more than one-third of Reddit users (n=37, 37%) did not disclose the type of brain tumor classification or gave an unclear description that could not be categorized (see Table 2).

**Table 1:** Categories of users among the top 100 Reddit posts under r/braintumor.

Classification of user	Users (n=88)^a^, n (%)
Writing about others with brain tumors	58 (66)
Parents	32 (36)
Spouse	9 (10)
Child	9 (10)
Extended family	1 (1)
Writing about self with a brain tumor	30 (34)

^a^Reddit users did not disclose their category in 12 posts and were omitted from the final percentage count.

**Table 2:** Brain tumor classification among the top 100 Reddit posts under r/braintumor.

Tumor type	Posts, n (%)
Glioblastoma	47 (47)
Unknown	37 (37)
Other primary brain tumor	16 (16)

### Qualitative Content Results

Lastly, the content analysis of these posts revealed how Reddit users primarily used the forum as a method to understand and process the emotions surrounding a brain tumor diagnosis. Three major themes within the content emerged: (1) harnessing hope, (2) appreciating the group, and (3) moving through the grief process.

Most of the posts of the subreddit “r/braincancer” were written by others who were witnessing the effects of brain tumors, specifically glioblastoma, on their loved ones. Our results are reasonable given that a majority of content that was posted was an in-depth storytelling experience in the treatment of the onset of a brain tumor with much less content focused on the end of life. Most of the Reddit users posted within a relatively short time, as the users were experiencing these events firsthand. One anonymous user wrote about their son, “At 4:44 this morning he took his last breath. He was at home with his wife and I by his side.” Other users allowed for some time to process before posting on Reddit. One user posted on Reddit about a week after receiving the news of their mother’s diagnosis and wrote:

They said there is no treatment they can offer us and she probably only has weeks to live. I am absolutely devastated and don't even know how to begin processing this.

The posting and real-life experiences occurred almost simultaneously, and the content topics revealed similar patterns of congruence following three major themes: (1) harnessing hope, (2) appreciating the group, and (3) moving through the grief process.

First, many of the Reddit users wrote encouraging words in the effort to *harness hope* for each other, and thereby, for themselves. The posts were generally written with an introductory description of their disease process, most notably by Reddit users who were also patients. Reddit users shared their own stories and acknowledged the severity of the disease. After this disclosure, many Reddit users then encouraged others in the forum. One user posted about their own diagnosis and then offered a suggestion to uplift others. They said:

Ever since being diagnosed with GBM Grade IV several months ago, I have gone through things I never imagined before the age of 24...But through all of it I have wanted to help other cancer patients by spreading what (little) I have learned about the whole process.

The user then described how making “silly” videos was therapeutic and ended the post by writing:

I know I am not the first person to do this, but I love making videos and being silly! So hopefully me putting up content about my surgery or mental health can help someone or someone you care about...Stay strong!

Other users went on to directly address the emotional sequalae of the brain tumor diagnosis and were direct in their encouragement to commend others to take hold of and harness their hope. A user who was diagnosed with anaplastic astrocytoma wrote:

So, if you're lost and feeling hopeless? Don’t be. It’s gonna be a long...journey filled with anxiety, scare or even depression. There will be tough days, but you gotta not let it bring you down every day you are alive after going through so much just to live.

Lastly, a vast majority of users concluded their posts by offering to be someone’s “listening ear” or to offer any support. One user who was 8 months from his diagnosis wrote “Keep fighting, everyone. If anyone has questions about my experience, I'm an open book.” These messages of resiliency remind other users to *harness hope* and move forward with bravery. The messages are a real-world account of lived experiences that are interwoven with the Reddit user’s own meanings of these experiences. This provides other users with applicable, comforting, and realistic messages tailored for a unique subgroup of a population dealing with brain tumor diagnoses.

Second, many of the Reddit users shared their *appreciation for the group*, which was most common among friends, caregivers, and siblings. Reddit users were thankful for the virtual support offered through the forum. After one user’s husband received a diagnosis of medulloblastoma, she was crippled by the prognosis and used Reddit to share and process her emotions. She candidly expressed her *appreciation for the group* by writing:

This subreddit, silly as it sounds, has been a God send...Even if no one reads this I want to thank you all for sharing here. I was alone and stunned sitting in my garage and not knowing what to do and you all gave me such insight and hope.

Another user wrote about their mother’s glioblastoma diagnosis and found comfort in the group setting. They wrote:

thank you so much to those kind souls who commented on here. I was feeling so alone and so overwhelmed and it honestly helped reading these.

In other cases, users were patients and expressed gratitude for the group while going through treatment. One man who was recently diagnosed with a brain tumor in his mid-20s described his clinical journey and concluded with:

So maybe I've been way off topic, but I really wanted to share my story. It's just really nice to talk about it. I rarely talk about this and have never shared my story on Reddit.

The supportive, nurturing environment of Reddit was acknowledged among both friends, caregivers, and family members as well as patients. Most of the users expressed their appreciation of the group with deep sincerity.

Third, several Reddit users (friends, caregivers, family members, etc) described their overwhelming emotions and the associated grappling as they *moved through the grief process* around their loved ones as explained by Kübler-Ross [24]. The content of these posts showed almost an equal representation of anger, depression (written by a variety of users), and acceptance, which was written exclusively by friends, caregivers, and family members. Little was mentioned surrounding denial or bargaining. One user posted about his wife who recently was diagnosed with glioblastoma. His anger was palpable. He wrote, “I want to punch someone. I want to break the terrariums and burn the wedding presents.” In the same post he went on to write:

I love her so much. How do you go on when your world crumbles? How do you eat? How do you focus on anything that even resembles the future? I hate the universe.

One woman wrote about her husband’s recent diagnosis and disclosed her painful realization about his prognosis and the associated depression. She said:

Knowing we'll never make that (anniversary)...It feels like all my hopes and dreams have shattered into a million pieces.

A son wrote about his father’s recent death and shared his depression:

I find myself extremely sad in waves. I'm ok for a little bit, but then think of something and I just cry. I'm not sure if that will go away.

Additionally, a patient with glioblastoma wrote about his depression as he moved through his own grief process and said:

...All I ever hear in my daily life is how good I look, or how inspiring I am. That couldn't be farther from the truth. I am a broken man just trying to do the right things.

Lastly, the voices of the Reddit users shifted to exclusively friends, caregivers, and family members as they wrote about the acceptance after a loved ones’ death. One user wrote about their father’s death from glioblastoma resembling some acceptance and said, “I am sad, we are all sad, but I am happy too, because he won't suffer through pointless treatment. He is free.” Another Reddit user posted about their father’s death and said:

I miss my dad a ton already, but we know that at least he got to see both his kids grown and married and had a good several years of retirement and travel with my mom to enjoy before he got sick.

## Discussion

### Principal Findings

In most of these posts, the users describe their emotional movement through the grief process with no expectation of a reply or comment from others. The posts, in turn, become a living therapeutic tool to help Reddit users process and heal from the emotional turmoil associated with brain tumors.

Overall, the three themes in the subreddit “r/braincancer” mirror the unique group psychotherapy process. Much of the content is aligned with Yalom’s [23] 11 therapeutic factors used in group psychotherapy, namely, installation of hope, universality, imparting information, altruism, group cohesiveness, catharsis, and existential factors. In the Reddit forum, users *harnessed hope* for themselves and others (Yalom’s [23] instillation of hope), and in this sharing, the users helped others to realize they are not alone in their journey (universality) with a brain tumor diagnosis. Additionally, users *harnessed hope* by offering advice and support to others (imparting information) thus creating therapeutic bonds within the forum members. Reddit users also were encouraging to one another (altruism) as they shared their *appreciation for the group*. This anonymous online forum created an overall feeling of acceptance and belonging (group cohesiveness) between members. Finally, Reddit users *moved through the grief process* where they shared their anger, depression, and acceptance (catharsis and existential factors; see Table 3).

**Table 3:** Overview of therapeutic factors and quotes in the top 100 Reddit posts under r/braintumor.

Yalom’s [23] therapeutic factors^a^	Definition	Quotes	Themes
Instillation of hope	Sharing good news, encouraging others, fostering hope	“So hopefully me putting up content [humorous videos] about my surgery or mental health can help someone or someone you care about...Stay strong!” “I've been struggling since the right side of my body went numb with my cognitive challenges but, we should stand strong. We are survivors until the day we die.” “Cancer is definitely a dark thing in our world and it's easy to drown in the misery that comes with it, but I would advise anyone going through this to ‘find their light’ in the situation and focus on that.” “There will be tough days, but you gotta not let it bring you down every day you are alive after going through so much just to live.”	Harnessing hope
Universality	Validating others’ experiences, helping members realize they are not alone	“Thank you so much to those kind souls who commented on here. I was feeling so alone and so overwhelmed and it honestly helped reading these.” “There are lots of support resources out there...you are not alone!”	Harnessing hope
Imparting information	Formally sharing knowledge, resources, ideas, advice	“But through all of it I have wanted to help other cancer patients by spreading what (little) I have learned about the whole process.” “If you have any questions or just want to talk, please PM me. Anything I can do to help. I love you all so much.” “...Get hospice involved early, you will need supplies, medicine, advice and help they can provide when you're lost.”	Harnessing hope
Altruism	Members finding meaning and value in listening and sharing in group	“Thank you so much to those kind souls who commented on here. I was feeling so alone and so overwhelmed and it honestly helped reading these.” “Hello everybody...Thank you to everyone who has offered their company, thoughts, and prayers with my SO [significant other] and I.”	Appreciation of the group
Group cohesiveness	Members feel gratitude for group; have a sense of acceptance, belonging	“I was alone and stunned sitting in my garage and not knowing what to do and you all gave me such insight and hope” “I can’t tell you how AMAZING each and every one of you are.” “This brain cancer stuff is really scary, and I just really appreciate the support of this group.” “I thank everyone for their support and comforting words over the past year.”	Appreciation of the group
Catharsis	Emotional release and promotes healing by sharing information to group	“I want to punch someone. I want to break the terrariums and burn the wedding presents.” “I hate that she has to go through this. I hate brain cancer. Just needed to rant for a second.” “I hate how glioblastoma takes away the person little by little.”	Moving through the grief process
Existential factors	Finding meaning through loss; life will continue on with pain, death, sadness, regret, joy	“I am sad, we are all sad, but I am happy too, because he won't suffer through pointless treatment. He is free.” “Taking the opportunity to reach out to those who have recently lost someone to this horrible disease and letting you know that day by day, things do get easier.”	Moving through the grief process

^a^Only seven of Yalom’s [23] therapeutic factors were applicable.

### Clinical Application and Comparison With Prior Work

The utility of social media platforms in brain tumor treatment and the associated outcome of palliative care continue to expand [25]. Our study is the first of its kind to evaluate the content within the public forum of Reddit. While our data did not necessarily reflect the transition to palliative care, this is a frequent progression for many patients with brain tumors, especially glioblastoma (almost half of our participants; see Table 2). As a result, HPs may want to consider assessing and treating associated symptoms, as these patients have high levels of anxiety, depression, and cognitive symptoms especially in transitioning to palliative care [26]. Additionally, psychological stress can also be a significant factor in the quality of survivorship in patients with brain tumors [27]. Other stresses included financial burden and workforce morbidity. These stressors affect patients from various sociodemographic backgrounds [28-30]. Rising out-of-pocket spending and drug costs increase the financial burden on patients [31]. One study showed that 40% to 80% of patients undergoing cancer treatment stop working [32], while other studies document that patients miss up to 6 months before returning to the workforce [33]. Online public anonymous forums such as Reddit may not necessarily offset the financial burden but may provide a way for patients to share their experiences or offer resources of additional financial support. All these factors are considerations in survivorship planning, and the Society for Neuro-Oncology Guidelines Committee has a recommended survivorship care plan for adult patients with brain tumors [4]. HPs may want to integrate social media or online, public, anonymous forums such as Reddit as a therapeutic tool into personalized care plans for caregivers as their loved ones enter into palliative care. HPs should consider telling patients and caregivers that, while such online forums can be virtual therapeutic spaces, patients and caregivers should still take precautions to safeguard against any unwanted spam or harmful posts (as with all online interactions). The moderation policies of this particular subreddit follow the eight guidelines listed for all of the Reddit communities, and moderators have the ability to ban any users who harass, bully, or promote violence [34].

### Limitations

While our analysis was unique in that we examined the content thread of the subreddit “r/braincancer,” our study was not without limitations. Our analysis only included the content of the original post. We attempted to set limits for our data analysis by focusing only on the original post. As a result, we did not include comments in our data analysis, and we may have missed more content and dynamic interactions within this virtual group setting. Our sample only included the top 100 upvoted (or recommended) posts, and these posts may not have been a full representation of the subreddit user experiences. Additionally, our data collection represents a point in time. Other posts may appear after data collection, as the subreddit posts are constantly evolving due to participant interactions. Additionally, the demographics of Reddit users skew towards young males [35], which might not reflect most patients and caregivers affected by brain tumors.

Researchers may want to consider analyzing all of the comments in the subreddit threads to better understand the group therapy processes within anonymous online forums such as Reddit. Additional qualitative analysis using the “best” filter may be another way to determine the interaction between participants. The “best” filter is an algorithm that adds weight to the quality of the vote based on the length of time the comment was posted. Using this feature may reveal additional posts to add another layer of richness to the data.

### Conclusions

The results of this study show the unique group therapy processes on a virtual platform for those affected by brain tumors. HPs may want to consider providing supportive online resources for their patients and caregivers. The various themes we identified in our sample are suggestions of topics that HPs may want to consider addressing to provide more tailored treatment planning.

## Abbreviations

HP: health care professional
NIH: National Institutes of Health
TRI: Translational Research Institute
